# Relationship Between Dietary Patterns and Blood Pressure During Adolescence: A Longitudinal Analysis From Dados Study

**DOI:** 10.1002/ajhb.24199

**Published:** 2024-12-29

**Authors:** Luis Miguel Fernández‐Galván, Mireia Adelantado‐Renau, Maria Reyes Beltran‐Valls, Diego Moliner‐Urdiales

**Affiliations:** ^1^ LIFE Research Group University Jaume I Castellon Spain

**Keywords:** cardiovascular disease, healthy food, hypertension, youth

## Abstract

**Background:**

Previous research in adults has suggested that healthy dietary patterns could be an effective strategy for blood pressure (BP) control. However, during adolescence, the scientific literature examining this relationship is scarce and controversial since inverse and null associations have been reported. Thus, the aim of our study was to analyze the relationship between the level of adherence to the Mediterranean diet (MD) and consumption of fresh fruits and vegetables at baseline with changes in BP over a two‐year period during adolescence.

**Methods:**

The analyses included 197 adolescents (92 girls) aged 13.9 ± 0.3 years. Adherence to the MD and consumption of fresh fruits and vegetables were assessed using the KIDMED questionnaire and a food frequency questionnaire, respectively. BP values were measured using an automatic sphygmomanometer according to standardized procedures.

**Results:**

Adolescents with high adherence levels to the MD, and high consumption of vegetables (but not fruits) at baseline, showed smaller increases in diastolic BP changes over two years (percentage of reduction ranging from ~48% to ~88%, *all p* < 0.026). No significant relationships were identified in systolic BP over the two‐year period.

**Conclusion:**

Our findings underscore the importance of promoting MD and vegetable consumption to reduce diastolic BP during adolescence, contributing to lowering future cardiovascular risk.

## Graphical abstract

Adolescents with high adherence level to the MD, high consumption of vegetables (but not fruits) at baseline, showed smaller increases in diastolic BP changes over a two‐year period (percentage of reduction ranging from ~48% to ~88%, *all p* < 0.026). No significant relationships were identified in systolic BP over the two‐year period.

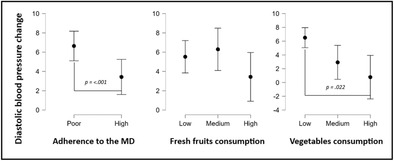

## 1 Introduction

Arterial hypertension is one of the main risk factors for cardiovascular disease and is the leading cause of death worldwide (Fuchs and Whelton 2020). In the adolescent population, normal blood pressure (BP) is defined as systolic BP (SBP) and diastolic BP (DBP) under the 90th percentile for age, sex, and height (Fuchs and Whelton 2020). According to data from the National Health and Nutrition Examination Survey (NHANES) (Rosner et al. 2013), there is an increase in the prevalence of high BP among adolescents (12% in girls and 19% in boys) between NHANES III (1988–1994) and NHANES (1999–2008), mainly induced by behavioral factors such as salt consumption, sedentary lifestyle, and unhealthy diet (Bubach et al. 2018).

A nonpharmacological treatment or prevention strategy for hypertension is based on the adoption of healthy eating habits (Cohen et al. 2017). In this sense, the Mediterranean diet (MD) is a traditional dietary pattern of the countries bordering the Mediterranean Sea, based on the consumption of olive oil, fruits, vegetables, whole grain cereals, nuts, and legumes. However, during the last decades, the dietary pattern adopted in these countries has changed, and for instance, more than 67% of Spanish adolescents have poor adherence to the MD (Grao‐Cruces, Fernández‐Martínez, and Nuviala 2014), and follow unhealthy dietary patterns characterized by consumption of foods with low nutritional value, especially those rich in sugars, saturated fats, and energy‐dense foods (Iaccarino Idelson, Scalfi, and Valerio 2017).

The changes toward unhealthy dietary patterns are relevant since they appear to be related to the occurrence of cardiovascular disease risk factors, like hypertension. Indeed, MD has been proven to be an effective dietary strategy in BP control in the adult population (Cowell et al. 2021) and adolescents (Mesas et al. 2022). However, the scientific literature examining the association between fruit and vegetable consumption and BP in adolescents is still scarce and reveals controversial results from cross‐sectional (Damasceno et al. 2011; Yang et al. 2018) and longitudinal studies (Rosário et al. 2018). For instance, Yang et al. (2018), who conducted a cross‐sectional study with a sample of Chinese children and adolescents, reported that daily consumption of at least three servings of vegetables was associated with a lower risk of hypertension. Similarly, in a sample of Brazilian adolescents Damasceno et al. (2011) concluded that those with higher levels of fresh fruit consumption showed lower levels of SBP and DBP, while those who regularly consume vegetables only had lower SBP levels. In addition, Rosário et al. (2018) reported in a longitudinal study that higher fruit consumption was associated with lower DBP only in adolescent girls, whereas no significant results were found for vegetable consumption.

Given that adolescence is a crucial stage in which major dietary habits are consolidated (Abbasnezhad et al. 2020), the aim of this longitudinal study was to analyze the relationship between the level of adherence to the MD and consumption of fresh fruits and vegetables at baseline with changes in SBP and DBP values at two‐year follow‐up during adolescence. We hypothesized that adolescents with a better dietary profile at baseline would have a smaller increase in BP during adolescence.

## 2 Materials and Methods

### 2.1 Study Design and Sample Selection

**FIGURE 1:**
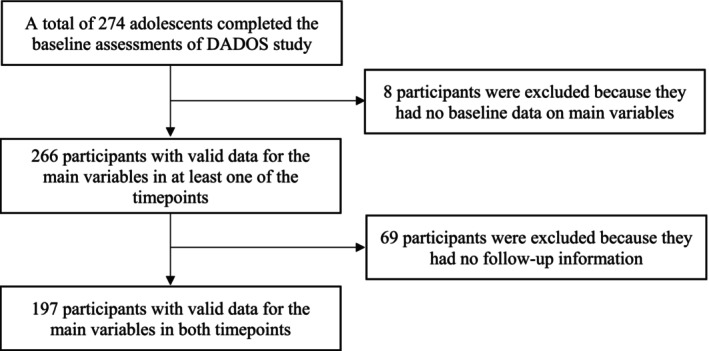
Flow chart of inclusion of participants.

The present study is part of the DADOS (Deporte, ADOlescencia y Salud) study, a 3‐year longitudinal research project aimed to investigate the role of physical activity in health, cognition, and psychological well‐being through adolescence. A convenience sampling technique was used to recruit participants. For that purpose, advertising leaflets about the research project were sent to secondary schools and sports clubs located in Castellon province (Spain), which included basic information about the objective of the study and the assessment protocol. Volunteers who met the inclusion criteria (i.e., to be enrolled in the second grade of secondary school and without diagnosed physical or neurological chronic diseases) were included in the study. Baseline data was obtained between March and June of 2015 for 274 participants, and follow‐up assessment was performed 2‐years later, between March and June of 2017 for 197 participants. Inclusion criteria for this study comprised having complete responses to dietary adherence questionnaires, consistent records of fruit and vegetable consumption, and accurate BP measurements at both time points. Figure 1 shows the flow chart of participants' inclusion. Posthoc power computation analyses, using the GPower 3.1 software (Faul et al. 2009), were performed on the primary outcomes of SBP and DBP. Based on BP‐specific means from the literature, an effect size of 0.25 (moderate) was set, with a significance level of α = 0.05. The analysis indicated that a total sample size of 169 participants would have been required to achieve a desired statistical power of 80% (1‐β = 0.80). The actual sample size of 197 participants used in the study aligns closely with this requirement, ensuring adequate sensitivity to detect significant differences between the two main groups (boys and girls).

Students and their parents or guardians were informed of the nature and characteristics of the study, and all provided written informed consent. The study protocol was designed in accordance with the ethical guidelines of the 1964 Declaration of Helsinki (last revision of Fortaleza, Brazil, 2013) and approved by the Research Ethics Committee of the Universitat Jaume I.

### 2.2 Adherence to the Mediterranean Diet

Adherence level to the MD was assessed using the KIDMED questionnaire (Serra‐Majem et al. 2004), which was based on the Mediterranean dietary guidelines for children and adolescents and provides an overall indication of their dietary patterns. The KIDMED includes 16 questions (12 with positive connotations and 4 with negative connotations) about the consumption of fast food, sweets, soft drinks, daily fruits and vegetables, and weekly fish and legumes, with yes or no answers required. Regarding the affirmative answers, a value of +1 was assigned to the questions with positive connotations in relation to MD (e.g., regular fruit consumption), while a value of −1 was assigned to the questions that constitute negative aspects (e.g., fast food consumption). Questions answered with “no” scored 0. The level of adherence to the MD was calculated as the sum of each answer, ranging from 0 to 12. According to the questionnaire protocol, the overall score was dichotomized as poor (0–7) or good (8–12).

### 2.3 Consumption of Fresh Fruits and Vegetables

Weekly consumption of fresh fruits and vegetables was assessed through the questions; How often do you eat fresh fruit? How often do you eat vegetables? Frequency was recorded as “rarely, less than 1 per day”, “usually, 1 per day”, and “very usually, more than 1 per day”. Based on the three answer options, the frequency of fresh fruits and vegetables consumption (separately) was categorized as “low”, “medium”, and “high” consumption.

### 2.4 Blood Pressure Measurement

According to standard procedures (McCrindle 2010), trained personnel measured seated BP in the right arm. After 5 min of rest in an isolated room with a comfortable temperature, SBP and DBP were assessed using an automatic sphygmomanometer (Omron HEM‐7321‐E, Omron Healthcare Co. Ltd., Kyoto, Japan), with an appropriate cuff size based on measured arm circumference. Two BP measurements were taken ≥ 2 min apart and the mean was used in the analysis. For the analyses, change in BP indicators was calculated as BP values at follow‐up minus BP at baseline.

To categorize participants, we applied the guidelines from the American Academy of Paediatrics (AAP) (Flynn et al. 2017), which established the same BP cutoffs for adolescents aged 13 and older as those for adults. According to these criteria, pre‐hypertension was defined as a SBP of 120–129 mmHg with a DBP < 80 mmHg, while hypertension was defined as SBP ≥ 130 mmHg and/or DBP ≥ 80 mmHg. These classifications were applied at both baseline and follow‐up to evaluate the prevalence of pre‐hypertension and hypertension and to analyze changes over the study period.

### 2.5 Covariates

Sex, pubertal stage, and body mass index (BMI) were included as covariates in the statistical analyses due to their relationship with the study variables (Cohen et al. 2017).

The pubertal stage was self‐reported using standardized pictures according to the five stages described by (Tanner and Whitehouse 1976), based on external primary and secondary sex characteristics. The level of development was assessed through two components: pubic hair growth for boys and girls, genital development in boys, and breast development in girls. The highest rating of the two components was used for the data analyses.

BMI was calculated as weight/height squared (kg/m^2^). Body weight was measured to the nearest 0.1 kg using an electronic scale (SECA 861—Hamburg, Germany). Body height was measured to the nearest 0.1 cm using a wall‐mounted stadiometer (SECA 213—Hamburg, Germany). Weight and height were measured in duplicate by trained staff following the standardized procedures and average measures were used for the data analyses.

### 2.6 Statistical Analyses

Descriptive characteristics of the study sample are presented as mean ± standard deviation or frequency (%). Differences between sexes and baseline and follow‐up measurements were examined by independent and paired samples t‐tests for continuous variables, respectively, and through chi‐squared test for contrasts of proportions. All variables were checked for normality and homogeneity using the Kolmogorov–Smirnov and Levene tests, respectively. Analyses of covariance (ANCOVA) with a Bonferroni posthoc test were conducted to examine whether changes in BP values over a two‐year period differed between dietary patterns categories (i.e., adherence to the MD and consumption of fresh fruits and vegetables). Analyses were adjusted for sex, pubertal stage, and BMI. Additionally, linear mixed models were employed to account for the repeated measures nature of the data, which enhances the robustness of our findings by accurately modeling the intra‐subject correlation and improving the precision of the estimates. The use of mixed models allowed us to manage potential correlations between repeated measurements on the same subjects and to adjust for time‐varying covariates, such as pubertal stage and BMI during the study period, thereby providing a more comprehensive understanding of the effects of dietary patterns on BP over time. All the analyses were performed using the Statistical Package for the Social Sciences (SPSS), v. 23 (IBM Corp., Armonk, NY, USA), and the level of significance was set to *p* < 0.05.

## 3 Results

A total of 197 adolescents aged 13.9 ± 0.3 years (47% girls) with valid data for adherence to the MD, frequency of fresh fruits and vegetables consumption, and BP at both baseline and follow‐up were included in the analysis. The participant selection process is shown in the flow chart included in Figure 1.

Table 1 shows descriptive characteristics of the study sample by sex at baseline and at two‐year follow‐up. Participants showed higher BP values at follow‐up compared to baseline (all *p* < 0.05). Girls showed lower SBP at follow‐up and higher DBP at baseline than boys (all *p* < 0.05). Participants showed higher consumption of fresh fruits and vegetables at follow‐up, and girls' consumption was higher than boys' at both baseline and follow up (all *p* < 0.05). In addition, 89 and 128 participants (45% and 65%) of the adolescents reported daily fresh fruits and vegetables consumption of < 1 time per day at follow‐up, respectively. Levels of adherence to the MD worsened over time showing lower levels in girls than in boys at baseline and at follow‐up (all *p* < 0.05). The prevalence of pre‐hypertension increased from 4 girls (4%) and 7 boys (7%) at baseline to 11 girls (12%) and 22 boys (21%) at follow‐up. Similarly, the prevalence of hypertension rose from 5 girls (5%) and 4 boys (4%) at baseline to 5 girls (5%) and 21 boys (20%) at follow‐up.

**TABLE 1:** Characteristics of the study sample by sex (*n* = 197).

	All (*n* = 197)		Girls (*n* = 92)		Boys (*n* = 106)	
Variables	Baseline	Follow‐up	Baseline	Follow‐up	Baseline	Follow‐up
Demographics						
Age (years)	13.9 ± 0.3	15.8 ± 0.3^a^	13.9 ± 0.3	15.9 ± 0.3^b^	13.9 ± 0.3	15.8 ± 0.3^b^
Pubertal stage (I‐V) (%)	0/8/32/50/10	0/0/10/52/38^a^	0/7/33/56/4	0/0/16/67/17^b^	0/9/31/44/16^c^	0/0/5/39/56^b,c^
Body mass index (kg/m^2^)	20.2 ± 2.7	21.8 ± 2.9^a^	20.6 ± 2.9	22.1 ± 3.2^b^	19.9 ± 2.5	21.5 ± 2.6^b^
Blood pressure						
Systolic (mmHg)	106.5 ± 10.7	113.6 ± 11.1^a^	105.5 ± 9.6	108.7 ± 9.0^b^	107.5 ± 11.6	117.7 ± 11.1^b,d^
Diastolic (mmHg)	64.9 ± 7.1	70.0 ± 6.1^a^	67.2 ± 7.2	69.8 ± 5.6^b^	62.9 ± 6.3^c^	70.3 ± 6.4^b^
Systolic difference (mmHg)	7.2 ± 10.2		3.4 ± 9.2		10.5 ± 9.8	
Diastolic difference (mmHg)	5.2 ± 8.5		2.6 ± 8.2		7.4 ± 8.1	
Pre‐hypertension (mmHg) *n* (%)	11 (6)	33 (17)	4 (4)	11 (12)	7 (7)	22 (21)
SBP 120–129 with a DBP < 80						
Hypertension; (mmHg) *n* (%)	9 (5)	26 (13)	5 (5)	5 (5)	4 (4)	21 (20)
SBP ≥ 130 and/or DBP ≥ 80						
Dietary patterns						
Adherence Mediterranean diet (0–12)	7.1 ± 2.2	7.2 ± 2.2	6.7 ± 2.2	6.7 ± 2.3	7.4 ± 2.3	7.6 ± 2.2
Poor; *n* (%)	106 (54)	109 (55)^a^	57 (62)	56 (61)	49 (47)^c^	53 (50)^bd^
High; *n* (%)	91 (46)	88 (45) ^a^	35 (38)	36 (39)	56 (53)^c^	52 (50)^bd^
Fresh fruits consumption						
Low (< 1 per day); *n* (%)	80 (41)	89 (45) ^a^	33 (36)	37 (40) ^b^	47 (44) ^c^	52 (50) ^bd^
Medium (1 per day); *n* (%)	60 (30)	42 (21) ^a^	30 (33)	22 (24) ^b^	30 (29) ^c^	20 (19) ^bd^
High (> 1 per day); *n* (%)	57 (29)	66 (34) ^a^	29 (31)	33 (36) ^b^	28 (27) ^c^	33 (31) ^bd^
Vegetables consumption						
Low (< 1 per day); *n* (%)	136 (69)	128 (65) ^a^	59 (64)	53 (57) ^b^	77 (73) ^c^	75 (72) ^d^
Medium (1 per day); *n* (%)	39 (20)	37 (19)	20 (22)	21 (23)	19 (18) ^c^	16 (15) ^bd^
High (> 1 per day); *n* (%)	22 (11)	32 (16) ^a^	13 (14)	18 (20) ^b^	9 (9) ^c^	14 (13) ^bd^

*Note:* Data are presented as mean ± standard deviation or frequency (%). Differences inter‐group, and intra‐group were examined by independent samples *t*‐test, and a paired samples *t*‐test, respectively. Differences in dietary patterns were examined by chi‐square test. (a) significant statistical differences over time in the total sample; (b) significant statistical differences over time by sex; (c) significant statistical differences at baseline between sex; (d) significant statistical differences at follow‐up between sex; all *p* < 0.05.

**FIGURE 2:**
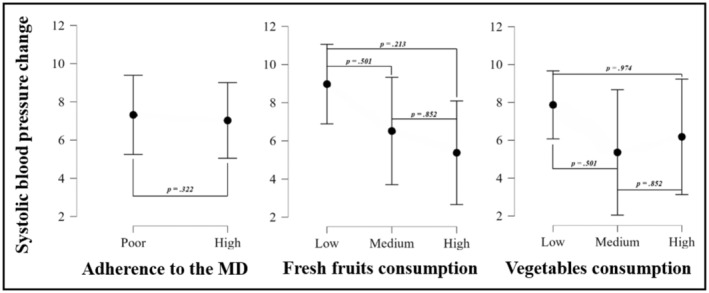
ANCOVA analyses showing differences in diastolic blood pressure change according to categories of different dietary patterns at baseline, after adjustment for sex, pubertal stage, and BMI at baseline. MD: Mediterranean diet. Levels of adherence were dichotomized as poor (0–7) and good (8–12). Fresh fruit consumption and vegetable consumption were categorized as follows: low (< 1 per day), medium (1 per day), and high (> 1 per day).

Figure 2 shows the change in SBP over two years according to categories of different dietary patterns at baseline, after adjusting for sex, pubertal stage, and BMI. No significant statistical differences were identified in SBP change among categories of adherence to the MD or fresh fruits and vegetables consumption.

**FIGURE 3:**
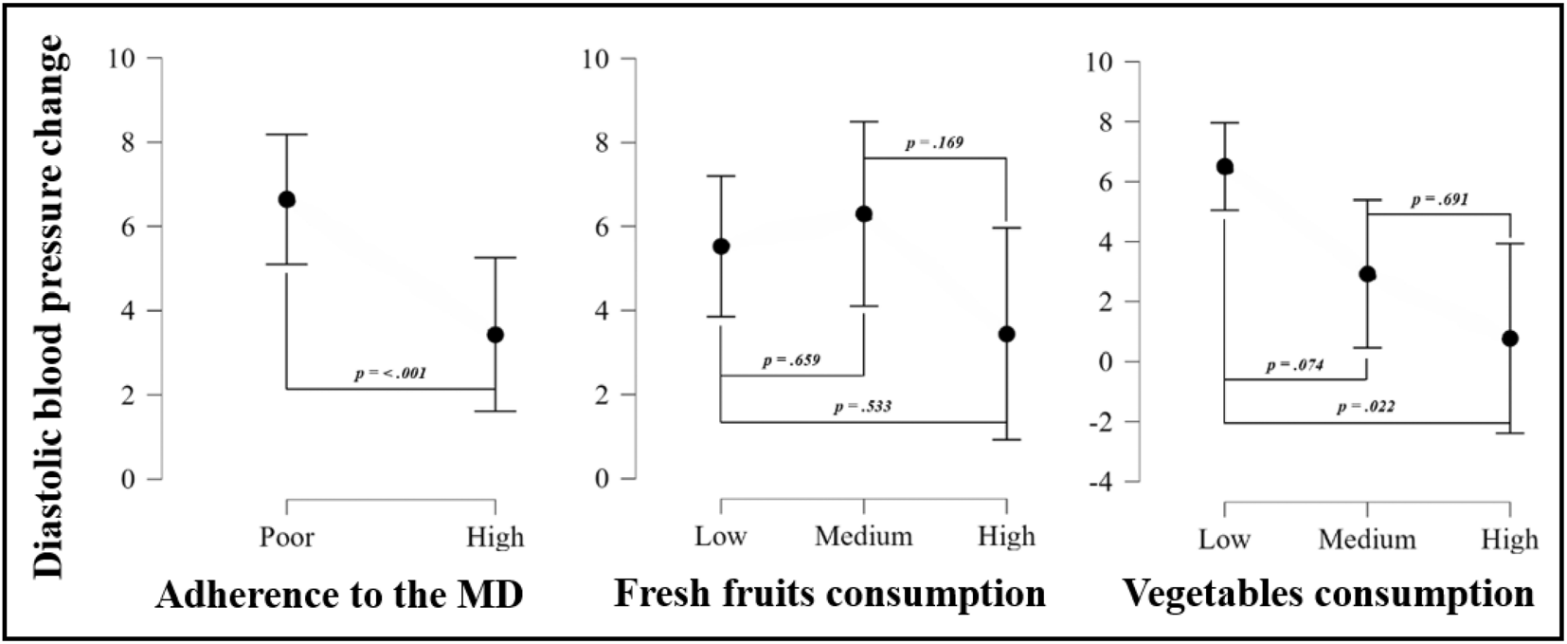
ANCOVA analyses showing differences in systolic blood pressure change according to categories of different dietary patterns at baseline, after adjustment for sex, pubertal stage, and BMI at baseline. MD: Mediterranean diet. Levels of adherence were dichotomized as poor (0–7) and good (8–12). Fresh fruit consumption and vegetable consumption were categorized as follows: low (< 1 per day), medium (1 per day), and high (> 1 per day).

Figure 3 displays the changes in DBP over two years according to categories of different dietary patterns at baseline, after adjusting for sex, pubertal stage, and BMI. Overall, adolescents with high levels of adherence to the MD showed a smaller increase in DBP (i.e., 48% decrease) than their peers with poor adherence to the MD (3.43 ± 0.92 vs. 6.64 ± 0.78; *p* ≤ 0.001). In addition, adolescents with high vegetable consumption showed a smaller increase in DBP (i.e., 88% decrease) compared to those with low vegetable consumption (0.77 ± 1.52 vs. 6.50 ± 0.74; *p* = 0.022).

Table 2 shows the association between dietary patterns at baseline and BP changes over a two‐year period, after adjusting for sex, pubertal stage, and BMI. No significant associations were found for SBP changes across any dietary pattern (*p* > 0.05). Adherence to the MD and daily vegetable consumption were significantly associated with smaller increases in DBP. Specifically, adherence to the MD showed a significant decrease in DBP (*B* = 4.03, *p* < 0.01), with a stronger association observed in girls (*B* = 4.11, *p* = 0.021) compared to boys (*B* = 3.85, *p* = 0.013). Similarly, daily vegetable consumption also showed a significant decrease in DBP (*B* = 2.65, *p* = 0.002) with a stronger association observed in girls (*B* = 2.94, *p* = 0.013) compared to boys (*B* = 2.39, *p* = 0.049). However, daily fresh fruit consumption did not show a significant association with DBP (*p* > 0.05) in either sex.

**TABLE 2:** Associations between dietary patterns at baseline and blood pressure change over a two‐year period.

	Systolic blood pressure					Diastolic blood pressure				
	*B*	*β*	95% CI	*t*	*p*	*B*	*β*	95% CI	*t*	*p*
Adherence to the Mediterranean diet	1.38	0.07	4.10; −1.36	0.99	0.322	4.03	0.24	6.29; −1.77	3.51	**< 0.01**
Girls	0.31	0.02	4.31; −3.70	0.15	0.879	4.11	0.24	7.58; 0.65	2.36	**0.021**
Boys	2.37	0.12	6.15; −1.41	1.24	0.217	3.85	0.24	6.87; 0.82	2.52	**0.013**
Daily vegetable consumption	0.65	0.04	2.63; −1.34	0.65	0.520	2.65	0.21	4.30; 1.01	3.17	**0.002**
Girls	0.23	0.02	2.89; −2.43	0.17	0.864	2.94	0.26	5.24; 0.65	2.55	**0.013**
Boys	1.11	0.07	4.12; −1.88	0.74	0.462	2.39	0.19	4.80; −0.03	1.96	**0.049**
Daily fresh fruit consumption	1.43	0.12	3.06; −0.21	1.72	0.086	0.66	0.07	2.06; −0.74	0.93	0.352
Girls	1.49	0.13	3.86; −0.88	1.25	0.214	1.34	0.13	3.45; −0.78	1.26	0.212
Boys	1.44	0.12	3.73; −0.85	1.25	0.215	0.27	0.03	2.16; −1.62	0.28	0.778

*Note:* Statistically significant values are presented in bold. All analyses were adjusted for sex, pubertal stage, and BMI at baseline.

Abbreviations: B, unstandardized regression coefficient; *β*, standardized regression coefficient.

## 4 Discussion

The main findings of our study suggest an inverse relationship during adolescence between healthy dietary patterns and DBP values at two‐year follow‐up. Adolescents with high levels of adherence to the MD and vegetable consumption showed lower increases in DBP values two years later. These results add new knowledge to the scarce previous literature examining the relationship between dietary patterns and BP in the adolescent population and contributes to the design of more effective nutritional strategies to prevent hypertension in youth.

In our sample, participants showed an increase in SBP and DBP over a two‐year period, this increase being higher in boys (*all p* < 0.05) than in girls. These results were expected since it is well known that in adolescence BP values increase progressively with body growth, mainly in boys, due to their later pubertal development and greater body mass (Wójcik et al. 2023).

Regarding dietary patterns, the results of our study showed that more than 50% of the participants had poor levels of adherence to the MD, which worsened over time. Our results agree with Serra‐Majem et al. (2004), who analyzed a sample of Spanish adolescents showing adherence to the MD of 53.6%, and with the study by Mesas et al. (2022) who found 62.9% of poor levels of MD adherence in Spanish adolescents. In terms of fruit and vegetable consumption, our study showed that between 45% and 65% of participants reported a daily consumption of fresh fruits and vegetables < 1 time per day during follow‐up, respectively. Or results are even worse than those found in the 2010 National Youth Physical Activity and Nutrition Study, where 28.5% and 33.2% consumed less than one serving of fresh fruits and vegetables per day, respectively (Centers for Disease Control and Prevention (CDC) 2011) and therefore fall far short of the World Health Organization recommendations, which recommend consuming at least 400 g of fresh fruits and vegetables per day (i.e., five portions per day).

Regarding BP values, results from both ANCOVA and linear mixed models analysis showed that adherence to the MD was inversely related to changes in DBP values, revealing lower increases in DBP at two‐year follow‐up among adolescents with high adherence to the MD compared to poor adherence to the MD. These results are in line with previous cross‐sectional studies showing that higher levels of adherence to the MD resulted in 42% and 37% less likely to present high BP or hypertension in adolescents (Mesas et al. 2022; So et al. 2013), respectively. This finding could be partially explained by the antioxidant effect of foods that are rich in high fiber, potassium, and magnesium content (Wang et al. 2014) commonly consumed in the MD, which may benefit BP during adolescence. In fact, these foods could reduce blood levels of inflammatory markers (Sureda et al. 2018), which could have an effect on weight control (Estruch and Ros 2020) or arterial stiffness (Lydakis et al. 2012), in turn resulting in healthier BP levels and better functioning of the cardiocirculatory system.

Concerning the consumption of fresh fruits and vegetables, we found lower increases in DBP at two‐year follow‐up among adolescents with a high level of vegetable consumption compared to those with low vegetable consumption when analyzed with both ANCOVA and linear mixed models analysis. These results are partly consistent with previous cross‐sectional research showing that higher levels of vegetable consumption were related to decreases in BP value (Yang et al. 2018). However, Damasceno et al. (2011) showed that vegetable consumption was only related to SBP. In our study, fresh fruit consumption was not related to changes in BP values, which partially agrees with the results found by Rosário et al. (2018), who reported statistically significant differences between fresh fruit consumption and DBP only in girls. Given that in younger people the most predominant BP abnormality is high DBP (Sundström et al. 2011), the results of our study could contribute to improving adolescents' cardiovascular health.

The analysis of changes in SBP values during the two‐year follow‐up did not reveal statistically significant relationships with the dietary patterns investigated. These data are similar to those reported by Collese et al. (2017), who reviewed five studies that examined the relationship between fresh fruits and vegetable consumption and SBP, showing that only one of them found a significant association between these variables (Damasceno et al. 2011). However, the mechanisms explaining why SBP is not related to diet patterns are unknown.

## 5 Limitations and Strengths

These results should be interpreted with caution due to some limitations. The consumption of fresh fruits and vegetables was not recorded as a quantitative value, which did not allow for analyzing differences among adolescents categorized as “high consumption”. In addition, although the analyses were controlled for several potential confounders, other variables such as consumption of salt, saturated fats, sugars, caffeine, or carbonated beverages, which were not considered in this study, could have influenced our results. Furthermore, while our study included a predominantly active sample, physical activity was not a primary focus and thus was not included in the analyses. Future studies should consider a broader and more heterogeneous sample in terms of physical activity levels. Additionally, we did not collect specific data on the total energy consumption or overall fiber intake of the participants, which could provide further insights into their dietary habits. We acknowledge that the use of convenience sampling may have compromised the representativeness of our sample, which could limit the generalizability of our findings and introduce potential biases. Future studies should consider a broader and more heterogeneous sample in terms of physical activity levels and include comprehensive dietary assessments. On the other hand, the main strength of this study was its longitudinal design with a homogeneous sample in terms of age, and the use of a validated questionnaire to assess adherence level to the MD (Serra‐Majem et al. 2004).

## 6 Conclusions

In conclusion, the results of this study suggest that high adherence to the MD and high consumption levels of vegetables were related to a lower increase in DBP over a two‐year period during adolescence. Our findings underscore the importance of promoting MD and vegetable consumption to reduce diastolic BP during adolescence, contributing to lowering future cardiovascular risk.

## Author Contributions

M.A.‐R., M.R.V.‐B., and D.M.‐U. revised the study, M.A.‐R., M.R.V.‐B., and D.M.‐U. conducted the data collection, L.M.F.‐G., M.A.‐R., and D.M.‐U. analyzed the data, L.M.F.‐G., M.R.V.‐B., and D.M.‐U. drafted the study and literature review. M.A.‐R., M.R.V.‐B., and D.M.‐U. revised the study critically for intellectual content and approved the final version to be published.

## Conflicts of Interest

The authors declare no conflicts of interest.

## Data Availability

The data that support the findings of this study are available from the corresponding author, Diego Moliner‐Urdiales, upon reasonable request.
